# Ozone Observations in Paris

**Published:** 1877-01

**Authors:** 


Ozone Observations in Paris.—Since 1865, ozone estimations have been continuously made at twenty different stations in Paris, of the results of which M. Marie Davy gives a diagrammatical summary in the monthly report of his observatory for May last. From this it appears that, while ozone abounds toward the periphery, and in the open parts of Paris, it is present only as a trace in the denser central quarters. The fact of the recognizable presence of ozone, even in the populous parts, is something to boast of. Much attention is being given to the photographic delineation of the microscopic particles of the atmosphere, as well as to the products of culture experiments with the living organic portion; and it looks like progress in the right direction to find a systematic table of "Matters contained in the air and rain" of each month, showing opposite each day, for the period of the day and night, the proportion, in 100 cubic metres of air, of ozone, carbonic acid, ammonia, nitric acid, saline residue, and organic matter. Similar systematic comparative information, collected at different points in the same city, will soon supply a solid structure of fact regarding its comparative hygienic conditions.—*Medical and Surgical Reporter.*

